# Inversely Estimating the Vertical Profile of the Soil CO_2_ Production Rate in a Deciduous Broadleaf Forest Using a Particle Filtering Method

**DOI:** 10.1371/journal.pone.0119001

**Published:** 2015-03-20

**Authors:** Gen Sakurai, Seiichiro Yonemura, Ayaka W. Kishimoto-Mo, Shohei Murayama, Toshiyuki Ohtsuka, Masayuki Yokozawa

**Affiliations:** 1 National Institute for Agro-Environmental Sciences, Tsukuba, Ibaraki, Japan; 2 National Institute of Advanced Industrial Science and Technology, Tsukuba, Ibaraki, Japan; 3 River Basin Research Center, Gifu University, Gifu City, Gifu, Japan; 4 Graduate School of Engineering, Shizuoka University, Hamamatsu, Shizuoka, Japan

## Abstract

Carbon dioxide (CO_2_) efflux from the soil surface, which is a major source of CO_2_ from terrestrial ecosystems, represents the total CO_2_ production at all soil depths. Although many studies have estimated the vertical profile of the CO_2_ production rate, one of the difficulties in estimating the vertical profile is measuring diffusion coefficients of CO_2_ at all soil depths in a nondestructive manner. In this study, we estimated the temporal variation in the vertical profile of the CO_2_ production rate using a data assimilation method, the particle filtering method, in which the diffusion coefficients of CO_2_ were simultaneously estimated. The CO_2_ concentrations at several soil depths and CO_2_ efflux from the soil surface (only during the snow-free period) were measured at two points in a broadleaf forest in Japan, and the data were assimilated into a simple model including a diffusion equation. We found that there were large variations in the pattern of the vertical profile of the CO_2_ production rate between experiment sites: the peak CO_2_ production rate was at soil depths around 10 cm during the snow-free period at one site, but the peak was at the soil surface at the other site. Using this method to estimate the CO_2_ production rate during snow-cover periods allowed us to estimate CO_2_ efflux during that period as well. We estimated that the CO_2_ efflux during the snow-cover period (about half the year) accounted for around 13% of the annual CO_2_ efflux at this site. Although the method proposed in this study does not ensure the validity of the estimated diffusion coefficients and CO_2_ production rates, the method enables us to more closely approach the “actual” values by decreasing the variance of the posterior distribution of the values.

## Introduction

The response of soil respiration to environmental change is of great concern, because an increase in atmospheric greenhouse gas concentrations and the subsequent temperature increase may stimulate the decomposition of soil organic carbon, and this stimulation may result in a strong positive feedback to global warming [1]. Therefore, it is important to improve our understanding of the characteristics of soil respiration. In particular, accurate knowledge of the response of soil carbon decomposition to changes in soil temperature is essential for reliable predictions of future carbon dioxide (CO_2_) emissions from terrestrial soils. Many field studies have examined the relationship between soil temperature and soil respiration [2, 3].

Estimating the vertical profile of soil respiration and its temporal variation is important for understanding the different responses to soil temperature among soil depths. Many previous studies have estimated vertical profiles of CO_2_ production rates [4–12]. However, in general, the diffusion coefficients that largely govern the estimated CO_2_ production rates are calculated in accordance with the soil temperature, soil water content, and gaseous phase ratio.

An unknown factor with a large effect on the CO_2_ production rates at different soil depths is the diffusion coefficient at each soil depth. Gas diffusion in soil is driven by molecular diffusion. The diffusion coefficients of soils are influenced by soil physical properties, including total porosity and water content [13]. Moreover, structures in the soil such as ant nests and plant roots could affect diffusion coefficients. However, it is difficult to continuously measure diffusion coefficients in a nondestructive manner. Therefore, when estimating the CO_2_ production rate, diffusion coefficients must be assumed to be constant throughout the observation period or are calculated based on soil porosity and soil moisture. Because soil water contents have large spatial variation [14], it may be the case that the water content measured near the site where the CO_2_ concentration is observed does not reflect the actual water content at the target point.

Our objective was to estimate the vertical profile of CO_2_ production rates while accounting for the uncertainty of the temporal and vertical variation of the soil diffusion coefficients. The temporal changes in CO_2_ production rates were estimated based on the vertical CO_2_ concentration profile. For this purpose, we applied a data assimilation method, the particle filtering algorithm [15–17]. Using observed CO_2_ concentration data, this Bayesian approach enabled us to update values for the diffusion coefficients of soil that were calculated by an empirical equation, like the Millington and Quirk equation [13], which estimates diffusion coefficients on the basis of soil temperature and moisture. First, we performed particle filtering to estimate the vertical profile and temporal variation of the soil CO_2_ production rate, while considering the uncertainty of the diffusion coefficient value. Next, we indirectly evaluated the validity of the method by comparing the CO_2_ effluxes estimated from CO_2_ concentration data alone with CO_2_ effluxes observed in the field. We also estimated the CO_2_ efflux during periods of snow cover, when CO_2_ efflux is difficult to measure.

## Materials and Methods

### Study site

The time-series data for soil-air CO_2_ concentrations were measured at the Takayama deciduous broadleaf forest site (36°08'N, 137°25'E; 1420 m a.s.l.) at the Takayama Field Station of the River Basin Research Center, Gifu University, located in Takayama City, Gifu Prefecture, in central Japan. A secondary forest consisting of oak (*Quercus crispula* Blume) and birch (*Betula ermanii* Cham.) trees with an understory of dwarf bamboo (*Sasa senanensis* [Franch. & Sav.] Rehder) covers this area. The soils at the site are Brown Forest Soils (Dystric Cambisols). The Oi and Oe horizons were 4–2 and 2–0 cm above the soil surface, and the A, AB, and B horizons were 0–17, 17–49, and 49–73 cm in depth, respectively (for details see Yonemura et al. [18]).

### Measurement of CO_2_ concentrations and other data

Yonemura et al. (2013) [18] reported the CO_2_ concentration data and results of simple analysis of these data from 10 June 2005 to 31 May 2006. For the measurement of soil CO_2_ concentrations, we used small nondispersive infrared (NDIR) CO_2_ sensors (GMP220 series; Vaisala, Helsinki, Finland). After holes were dug with an auger, the CO_2_ sensors were inserted vertically into the soil at depths of 5, 10, 20, and 50 cm at locations adjacent to the open-flow chambers used to measure the soil surface CO_2_ efflux [19] (therefore, the surface and underground sensors were not exactly co-located). Pairs of CO_2_ sensors were installed at 5, 10, and 20 cm. We used daily averaged data in our analyses. The target period was from 10 June 2005 to 31 December 2007 (935 days). Soil CO_2_ concentrations were measured at two locations (points A and B) about 5 m apart. At point A, soil CO_2_ was measured at depths of 0, 5, 10, 20 and 50 cm; at point B, soil CO_2_ was measured at depths of 0, 5, 10, and 20 cm.

The top of each CO_2_ sensor (that is, the interface between the air in the NDIR sensor chamber and the soil air) was covered with a vinyl chloride sheath custom-made by Sensor Co. Ltd. (Inagi, Tokyo, Japan) to protect the sensors, principally against water. The CO_2_ sensor output is an electrical current between 40 and 200 mA. Precision resistors (100 Ω) attached to the CR10X datalogger (Campbell Scientific, Logan, Utah, USA) were used to convert the current into a voltage between 400 and 2000 mV.

The responses of NDIR sensors are known to drift with the passage of time [20]. To detect this drift, a pipe was inserted into a sheath attached to each CO_2_ sensor so that CO_2_-free air or a standard gas of known CO_2_ concentration could be introduced into the buried sensors for calibration. As CO_2_ standard gases, we used air containing 1569, 9047, or 16,130 ppm CO_2_ (Takachiho Kagaku Kogyo, Tokyo, Japan). About 3 to 10 minutes were needed to obtain enough reliable data for calibration after introducing a standard gas into the CO_2_ sensors. The calibration procedure was conducted on 10 June 2005, 16 September 2005, and 20 June 2006, and a linear calibration relationship was determined after pressure and temperature corrections.

The execution interval of the data logger was set to 10 min, and data were recorded at intervals of 10 or 30 min. We used daily averaged data in our analyses. We also compared the air temperature inside each sensor with the soil temperature at the sensor's depth and found that they agreed to within 1°C, indicating that any temperature increase due to the sensor's presence in the soil surrounding the sensor was local and small. In this study, the CO_2_ sensor was not switched off in order to guarantee the absence of water condensation in the long-term—from our experience, water condensation causes the CO2 sensors to malfunction. Temperature increase in ambient soil were considered to be small in this study because of the heat resistance between CO_2_ sensors through the vinyl chloride sheath and because of constant temperature control of the CO_2_ sensor.

Soil CO_2_ efflux data at the soil surface were obtained by batch measurements using a flow-through chamber [19], except during periods that included snow cover (efflux was not measured from 5 November 2005 to 8 May 2006, from 25 November 2006 to 24 May 2007, and from 23 November 2007 to 31 December 2007). For the soil CO_2_ efflux data, we used the averages of values from a line of four chambers spaced about 5 m apart. Measurements of other environmental data at the site were described in detail elsewhere [21,22].

### Model

For inverse estimation of the values relevant to the diffusion coefficient and CO_2_ production rate, we assumed a simple one-dimensional diffusion equation for the CO_2_ transport [10], as follows:

$$\frac{\partial C\left(t,z\right)}{\partial t}=\frac{\partial }{\partial z}\left(D_{\text{S}}\left(t,z\right)\frac{\partial C\left(t,z\right)}{\partial z}\right)+S\left(t,z\right),$$ (1)

where *C*(*t*, *z*) is the gas-phase CO_2_ concentration (mol m^−3^) within the soil pore space at time *t* and depth *z* below the soil surface, *D*_s_(*t*, *z*) is the soil gas diffusivity (m^2^ day^−1^), and *S*(*t*, *z*) is the CO_2_ production rate (mol m^−3^ day^−1^). The maximum soil depth simulated was 60 cm and the minimum soil layer was −1 cm (i.e., 1 cm above the soil surface); the range was separated into 62 1-cm layers (from −1 to 60 cm). For the boundary condition of the layer immediately above the soil surface (−1 cm), we assumed a Dirichlet boundary [23] condition in which the CO_2_ concentration was set to the atmospheric CO_2_ concentration. For the boundary condition of the deepest layer (60 cm), we assumed a Neumann boundary condition [23] in which the CO_2_ concentration of the layer at 59 cm is equal to that at 60 cm. The differential equations were solved using an implicit method with a daily time step.

### Particle filtering

We applied a particle filtering algorithm [15–17] for the inverse estimation of the CO_2_ production rate and the diffusion coefficient at each soil depth and each time step. The particle filtering algorithm is a general Monte Carlo sampling method for performing inference in state-space models in which the state of the system evolves over time. In this algorithm, the posterior distributions of the target values (state variables) at time *t* are sequentially estimated using Bayesian statistics, and the probability distributions of the state variables at time *t−*1 are used as the prior distributions. The probability distributions of the state variables are approximated by a large number of particles.

When using the particle filtering algorithm, the system equation and observation equation must first be defined. The system equation is the statistical model that describes the transition process of state variables from time *t*—1 to time *t*. The observation equation is the statistical model that describes the relationship between the state variables and the observation variables. At time *t*, we generate a proposal distribution from the distributions of the state variable at time *t−*1 according to the system equation. We then estimate the observation variables according to the observation equation. Each particle is weighted according to the likelihood calculated based on the observed data at time *t*. By resampling the particles according to the weightings, we obtain the posterior distributions of the state variables at time *t*. The temporal changes of the state variables can be obtained by repeating this procedure.

In this study, the system and observation equations were defined, respectively, as follows:

Q(t)=Q(t−1)+e(t), (2)

Y(t)=F[C(t−1),Q(t)]+s(t), (3)

where **Q**(*t*) is the set of state variables at time *t*, **e**(*t*) is a system noise vector that follows a multivariate normal distribution with variance-covariance matrix Σ_s_, **C**(*t−*1) is a vector that includes the CO_2_ concentrations (mol m^−3^) of the 62 soil layers at time *t*, **s**(*t*) is an observation error vector that follows a multivariate normal distribution with variance-covariance matrix Σ_o_, **F** is the function that calculates the CO_2_ concentrations and CO_2_ efflux (mol m^−2^ day^−1^) from the soil surface at time *t* according to Equation (1), and **Y**(*t*) consists of 63 components that comprise the CO_2_ concentration in each soil layer and the CO_2_ efflux value at each soil surface. For simplicity, the nondiagonal elements of the factors Σ_s_ and Σ_o_ are set to zero, which means that the errors are independent of each other. The number of particles was set to 10,000.

### Estimated variables

We estimated the CO_2_ production rates and diffusion coefficients as state variables, using the particle filtering algorithm to estimate the diffusion coefficients at six soil depths (−1, 0, 15, 30, 45, and 60 cm). We assumed that the values of diffusion coefficients decrease with depth [10] and interpolated the values using cubic spline smoothing at the −1 cm layer. We estimated the CO_2_ production rates at seven soil layers (0, 5, 10, 20, 35, 50, and 60 cm), and the CO_2_ production rate of each soil layer was interpolated using cubic spline smoothing. Therefore, we estimated the posterior distribution of the 13 state variables (six for diffusion coefficients and seven for CO_2_ production rates) at each time step. During the period when the CO_2_ efflux was not measured (mainly during snow-cover periods), the maximum CO_2_ production rate and diffusion coefficient values were set at the averages of the estimated maximum values over the period without snow cover in 2005 for each soil layer.

Because we did not have any information about the observation error distribution, we also estimated values of the diagonal elements of Σ_o_ as time-independent parameters [15]. For this estimation, we assumed that the standard errors of the error distributions of CO_2_ concentration and CO_2_ efflux were proportional to the respective absolute values of the observations. Thus, in addition to 13 state variables, we estimated two more time-independent parameters: the proportional constants for CO_2_ concentration and CO_2_ efflux.

### Initialization and error distribution

To set the initial values of the 13 state variables, we conducted a spin-up procedure in which the initial values of the diffusion coefficients and CO_2_ production rates were estimated based on the assumption that ∂**C**(1)/∂*t*+0. The CO_2_ concentration in each soil layer at *t* = 1 was interpolated using cubic spline smoothing.

The values of the diagonal elements of Σ_s(CO2)_, which represents the standard deviation of the proposal distribution from **Q**(*t−*1) to **Q**(*t*) for the CO_2_ production rate, were set to 0.68, 0.42, 0.15, 0.057, 0.03, 0.016, and 0.01 mol m^−3^ day^−1^, for 0, 5, 10, 20, 35, 50, and 60 cm respectively; these are average values at the respective depths for diurnal changes in CO_2_ production rates estimated by Yonemura et al. [18]. In that study, the CO_2_ production rates were estimated by using a simple equation [24] that assumed a uniform distribution of diffusion coefficients, but the values were estimated using data obtained at the same site.

The values of diagonal elements of Σ_s(D)_ for the diffusion coefficient were estimated using soil temperature and water content data according to the equation used by Yonemura et al. [10], which assumed a simple relationship between soil water content and the diffusion coefficient [13]. Using these estimated diffusion coefficient values, we calculated the average absolute values of the diurnal change of the diffusion coefficients at each soil depth (5, 15, and 40 cm). Because water content was measured at only three soil depths, we estimated the standard deviation of the proposal distribution for diffusion coefficients at soil depths of 0, 15, 30, 45, and 60 cm by applying linear regression to the three soil depths. The resulting estimated diagonal elements of Σ_s(D)_ for the diffusion coefficients were 0.05, 0.04, 0.03, 0.03, 0.02, and 0.01 m^2^ day^−1^ for soil depth of −1, 0, 15, 30, 45, and 60 cm, respectively. Because this procedure uses soil water content and employs a linear regression of three points at the level of daily changes in the diffusion coefficients obtained from the calculations, a high level of accuracy is probably not assured. However, these values are averages of system noise; the diffusion coefficients are related only very indirectly to CO_2_ production rates at the respective times. The magnitude of system noise is primarily related to predictions made for times when study data are not available. In this study, it is likely that system noise did not have a particularly large effect on the estimated values of the parameters.

### Likelihood calculations

In the particle filtering procedure, we calculated the likelihood for each particle (parameter set) for each time step using observed soil CO_2_ concentration and CO_2_ efflux from the soil surface. However, because this method seeks to determine both the diffusion coefficients and the CO_2_ production rates simultaneously, it may be difficult to identify unique solutions. Therefore, in the likelihood calculation we also considered the values of the diffusion coefficients calculated for each day from the soil temperatures and soil water contents. That is, for each of the three depths at which the soil water content was measured (5, 15, and 40 cm), we calculated the diffusion coefficient for each day using the equation of Millington and Quirk [13] and used this value in the likelihood calculation together with the CO_2_ concentration and CO_2_ efflux. This change yields estimated diffusion coefficients that are closer to those calculated in accordance with Millington and Quirk [13]. In other words, the diffusion coefficients that were calculated using the Millington and Quirk equation were updated using the observed CO_2_ concentration data through the particle filtering procedure, in which the result of the Millington and Quirk equation was used as the “prior information”. Therefore, changes in the diffusion coefficients caused by factors not considered by Millington and Quirk [13] (that is, factors other than soil temperature and water content) are reflected to some extent by the inverse estimation with the particle filter. We imposed the additional limitation that the diffusion coefficients decrease at greater depths in the soil when the previously calculated diffusion coefficients from the Millington and Quirk equation show the same tendency. For the variance of the error distribution of the likelihood calculations, we used the variance of the diffusion coefficients calculated using the Millington and Quirk equation.

In summary, for the particle filter we used three kinds of values in the likelihood calculations: values calculated from the CO_2_ efflux through the soil surface (in snow-free periods only); field measurements of CO_2_ concentrations in the soil; and diffusion coefficients calculated in accordance with Millington and Quirk [13].

### Validation testing

We checked the level of reliability of the model outputs that were estimated by our method in the following manner. Where efflux data from the soil were not used as study data (not used in the likelihood calculation), we investigated how well the efflux of CO_2_ from the soil could be inferred. In other words, we checked to what extent the efflux from the soil could be inferred only from the measurements of CO_2_ concentrations in the soil. To this end, at each of points A and B, we compared CO_2_ effluxes provided by the model assimilating two kinds of data—the CO_2_ measurements and the calculated diffusion coefficients—with the observed CO_2_ effluxes from the soil.

### Experimental data for temperature sensitivity

For further indirect confirmation of the validity of the model estimation, we compared the temperature sensitivities of the CO_2_ production rate estimated by the particle filtering method with those estimated experimentally. For our measurements of CO_2_ production rate, we sampled the soil from depths of 0–10 cm, 10–20 cm, and 20–40 cm at the observation site, then put the samples through a 2 mm sieve and placed them in 263 ml bottles (three replicates for each soil depth). The bottles were incubated at 5, 15, 25, and 35°C for 30 days, during which time the CO_2_ concentrations of the gas in each bottle were measured every three or four days. The soil moisture was fixed at 60%. The CO_2_ production rates were calculated by the mass balance equation. The number of replicates is was three for each temperature setting.

### Calculation of temperature sensitivity

The temperature sensitivity of the resulting CO_2_ production rates was estimated with the following equation:

$$f\left(T\right)=aQ^{\frac{T}{10}}+bW,$$ (4)

where *a* and *b* are parameters, *T* is soil temperature, *W* is soil water content, and *Q* is Q_10_ value. For the experimental data, *W* was set at zero because the soil water content did not change. For the estimation of the Q_10_ values from our incubation experiments in the laboratory, we calculated the Q_10_ values by using a bootstrap sampling method in which the CO_2_ emission rates were randomly sampled from the results of the three replicate lines and Q_10_ values were calculated for that sample set. This procedure was repeated 10,000 times, and the average and standard error of the resulting set of Q_10_ values were estimated for each depth. For field data, we estimated the temperature sensitivity at 5, 15, and 40 cm depths for comparison with the experimental data because we had data on water contents only for these depths.

We used R version 2.15.3 [25] for the simulation and analysis. The Q_10_ values were estimated using the “optim” function of R. The R code for our analysis is available in the S1 Sample Code.

## Results

**Fig 1:**
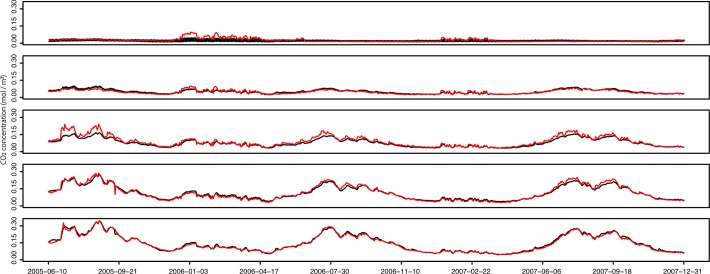
Observed and estimated CO_2_ concentration at 5 depths at point A. The red lines and black lines show observed and estimated CO_2_ concentrations, respectively, at 0, 5, 10, 20, and 50 cm soil depths from 2005 to 2007 at point A. The estimated values were the average of 10,000 particles for each time step.

The estimated soil CO_2_ concentrations replicated well the observed CO_2_ concentrations for all soil depths. Fig. 1 shows the observed and estimated temporal changes in CO_2_ concentrations at the five soil depths of point A. The root-mean-square errors (RMSE) between the observed and estimated CO_2_ concentrations were 0.017, 0.009, 0.018, 0.011, and 0.009 mol m^–3^ for 0, 5, 10, 20, and 50 cm soil depths, respectively, for point A and 0.017, 0.028, 0.015, and 0.022 mol m^–3^ for 0, 5, 10, and 20 cm soil depths, respectively, for point B.

### Vertical profile of CO_2_ production rate

**Fig 2:**
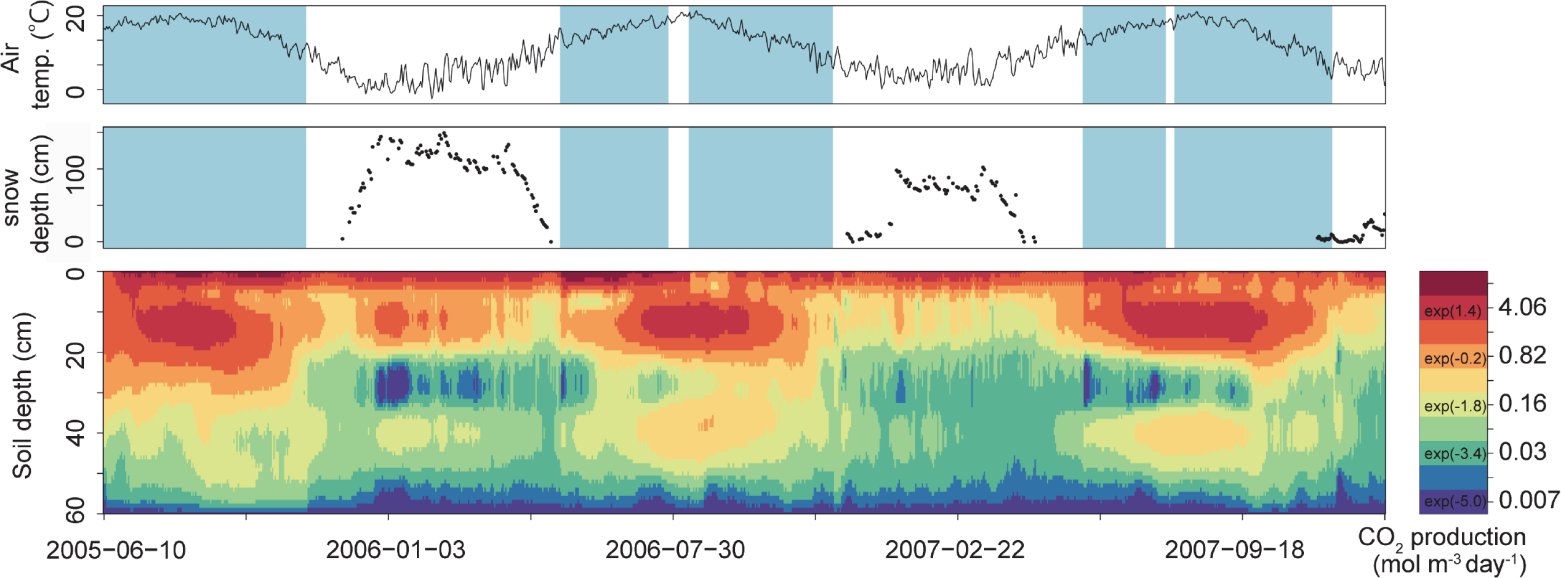
Observed air temperature, snow depth, and estimated CO_2_ production rates at point A. The top, middle, and bottom plots show observed air temperature at 10 m above the soil surface (°C; top), observed snow depth (cm; middle), and estimated CO_2_ production rates (mol m^−3^ day^−1^; bottom) at point A, respectively. The CO_2_ production rates (described by a natural logarithmic expression) are illustrated by the color gradient. Blue shaded areas indicate the period when CO_2_ efflux from the soil surface was measured.

Fig. 2 shows the average estimated CO_2_ production rate for each time step and each soil depth, together with the observed air temperature (10 m above the soil surface) and snow depth (cm) for point A. For the entire experimental period, the average CO_2_ production rate at 0, 10, 20, 30, 40, 50, and 60 cm soil depth was 3.38, 1.28, 0.51, 0.14, 0.16, 0.068, and 0.0049 mol m^–3^ day^–1^, respectively. For the periods when the CO_2_ efflux was measured (mainly snow-free periods), the average values were 3.76, 1.84, 0.81, 0.21, 0.21, 0.086, and 0.0050 mol m^−3^ day^−1^, respectively, with the peak rates occurring around 0 and 10 cm (Fig. 2). During the periods when the CO_2_ efflux was not measured (mainly snow-cover periods), the average CO_2_ production rates were 2.93, 0.62, 0.16, 0.051, 0.10, 0.047, and 0.0047 mol m^−3^ day^−1^, respectively.

**Fig 3:**
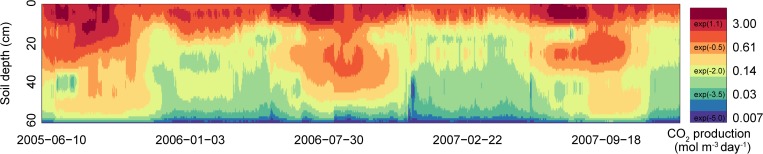
Estimated CO_2_ production rates (mol m^–3^ day^–1^; bottom) at point B. The CO_2_ production rates (described by a natural logarithmic expression) are illustrated by the color gradient. Blue shaded areas indicate the period when CO_2_ efflux from the soil surface was measured.

Fig. 3 shows the average estimated CO_2_ production rate for each time step and each soil depth for point B. For the entire experimental period, the average CO_2_ production rate at 0, 10, 20, 30, 40, 50, and 60 cm soil depth was 1.94, 0.91, 0.47, 0.35, 0.19, 0.14, and 0.0049 mol m^−3^ day^−1^, respectively. For the periods when the CO_2_ efflux was measured (mainly snow-free periods), the average values were 2.49, 1.17, 0.71, 0.52, 0.27, 0.19, and 0.0051 mol m^–3^ day^−1^, respectively, with the peak rates occurring only around 0 cm. During the periods when the CO_2_ efflux was not measured (mainly snow-cover periods), the average CO_2_ production rates were 1.30, 0.60, 0.18, 0.15, 0.10, 0.080, and 0.0047 mol m^−3^ day^−1^, respectively.

The estimates of CO_2_ production rates in the soil have some uncertainty. This uncertainty may be expressed by using a data assimilation technique. For some of the time steps, averages and 95% confidence intervals for the CO_2_ production rates calculated for the respective time steps are shown in the S1 Fig. and S2 Fig.

### Estimated CO_2_ efflux

**Fig 4:**
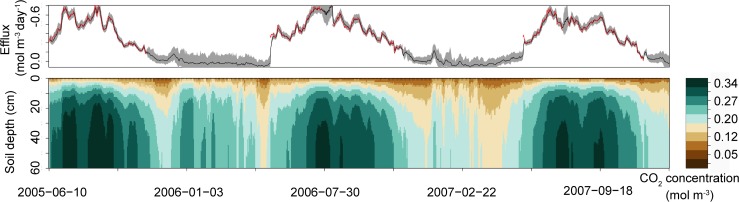
Observed and estimated CO_2_ effluxes and estimated CO_2_ concentration at point A. The red and black lines (top) show observed and estimated CO_2_ effluxes (mol m^–2^ day^–1^) from the soil surface, respectively. The shaded area indicates the 95% confidence interval. The bottom plot shows estimated CO_2_ concentration (mol m^–3^; bottom) at point A. The estimated values are the average of 10,000 particles for each time step. The CO_2_ concentrations are illustrated by the color gradient.

The average estimated CO_2_ effluxes from the soil surface and average estimated CO_2_ concentrations are shown in Fig. 4 for point A. During the snow-cover periods, the estimated average daily CO_2_ effluxes were −0.086 (±0.0032 SD) and −0.087 (±0.0029) mol m^–2^ day^–1^ during the winters of 2005–2006 and 2006–2007, respectively. The cumulative CO_2_ effluxes from the soil surface during snow-cover periods were −13.03 (±0.49) and −11.95 (±0.40) mol m^−2^ during 2005–2006 (152 days) and 2006–2007 (137 days), respectively, which were equivalent to 14% and 12% of the cumulative CO_2_ efflux from the soil surface during the whole experimental period in 2005–2006 (beginning from 10 June) and 2006–2007, respectively. For point B, the estimated average daily CO_2_ effluxes were −0.096 (±0.0041 SD) and −0.085 (±0.0032) mol m^−2^ day^−1^ during the winters of 2005–2006 and 2006–2007, respectively. The cumulative CO_2_ effluxes from the soil surface during snow-cover periods were −14.52 (±0.63) and −11.60 (±0.43) mol m^−2^ during 2005–2006 and 2006–2007, respectively, which were equivalent to 15% and 11% of the cumulative CO_2_ efflux from the soil surface during the whole experimental period in 2005–2006 (beginning from 10 June) and 2006–2007, respectively.

### Model validation

**Fig 5:**
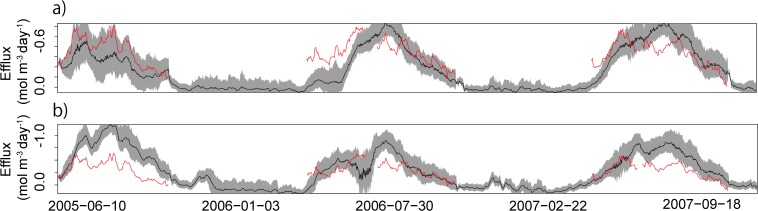
Observed (red) and estimated (black) CO_2_ (mol m^−2^ day^−1^) from the soil surface for point A (a) and point B (b). In this figure, the CO_2_ effluxes were estimated only using the data about CO_2_ concentration in the soil. The shaded area indicates the 95% confidence interval.

Fig. 5 shows the estimated CO_2_ effluxes from the soil surface in which the effluxes were estimated using only the data for CO_2_ concentrations in the soil. The RMSE between estimated and observed CO_2_ effluxes were 0.054 mol m^−2^ for point A and 0.16 mol m^−2^ for point B.

### Temperature sensitivity

The estimated Q_10_ values for the field data were 1.67, 2.23, and 2.54 for soil depths of 5, 15, and 40 cm, respectively for point A and 2.25, 2.47, and 2.67 for soil depths of 5, 15, and 40 cm, respectively for point B. The estimated Q_10_ values from our incubation experiments in the laboratory were 2.26 (±0.022 SD), 2.63 (±0.028 SD), and 2.37 (±0.076 SD) for soil depths of 0–−10, 10–20, and 20–40 cm, respectively.

## Discussion

Previous estimates of the vertical profile of CO_2_ production rates have been mainly based on soil diffusion coefficients estimated using soil temperature and water content data [4–12]. However, the total porosity in soil can vary during the observation period for the CO_2_ concentrations, and the vertical variation of soil water content is quite large, making it difficult to interpolate the soil water content to all target depths from data measured at only a few soil depths. In addition, factors other than soil temperature and water content, such as changes in the structure of ant nests, the structure of plant roots, and the distribution of soil organisms (e.g., earthworms), may also affect the diffusion coefficients. Therefore, in this study, we estimated the temporal variation of diffusion coefficients at each soil depth together with the CO_2_ production rates using a particle filtering method.

There have been many studies estimating the vertical distribution of soil CO_2_ production rates [4–12] and studies estimating soil diffusion coefficients [6,7,26,27]. In those studies the diffusion coefficients were estimated by using soil temperature and soil water content. However, estimated values of diffusion coefficients that are calculated from soil temperatures and water contents differ greatly depending on the equation used for finding the diffusion coefficients, and they have significant uncertainty [6,7,26]. The particular feature of this study is simultaneous inverse estimation by a data assimilation method of CO_2_ production rates and soil diffusion coefficients with a spatial resolution of 1 cm and a temporal resolution of 1 day. By the application of this method, the dynamics of CO_2_ production rates can be estimated in detail and at the same time levels of uncertainty can be estimated.

Currently, CO_2_ concentrations can be estimated automatically at relatively low cost, making it feasible to measure soil CO_2_ concentrations at multiple soil depths and multiple locations (spatial points). If data on soil CO_2_ concentrations can be collected both at multiple soil depths and at multiple spatial points, future studies should be able to estimate CO_2_ production rates in three dimensions by applying this method. This kind of research promises to advance our understanding of the relationship between CO_2_ production and environmental conditions.

### Vertical profile of the CO_2_ production rate

For point A, the estimated CO_2_ production rates were greatest around 0 and 10 cm during snow-free periods and around the soil surface during the snow-cover periods (Fig. 2). According to previous studies, the estimated CO_2_ production rates were highest only around the soil surface [4–12]. One factor that could cause the difference in the estimated peaks of the CO_2_ production rate between the present and previous studies is the difference in the resolution of the simulated soil depths. In most of the cited studies, the maximum soil depth was deeper than that in the present study but the resolution was lower. We measured CO_2_ concentrations at 0, 5, 10, 20, and 50 cm soil depths, which allowed us to estimate the CO_2_ production rate around the soil surface with high resolution.

The maximum CO_2_ production rates were found around 10 cm at site A and maybe due to root respiration, mainly that of dwarf bamboo. Previous studies estimated the contribution of root respiration to total soil respiration to be around 40% in a temperate forest [28] and around 45% in a cool-temperate forest [29]. The dwarf bamboo roots were mainly distributed from 0 to 10 cm in the soil, and this would explain the large production of CO_2_ around 10 cm during the snow-free period. However, the relationship between root biomass and CO_2_ production is not be clear [30–32]. Future studies are needed to estimate the actual contribution of root respiration to the vertical profile of CO_2_ production rates, for example, by comparing the vertical profiles in various soils that have different root distributions.

The variability of the water content near the soil surface may explain why the CO_2_ production rates near the soil surface were low during the hottest season (around August) (Fig. 2). Although we did not measure the water content near the soil surface, it generally has great variability, and dry or very moist conditions can cause low respiration rates in soil microorganisms [8]. The high CO_2_ production rate near the soil surface during the snow-cover period (Fig. 2), during which soil moisture near the soil surface would have low variability, may indirectly support this hypothesis.

At point B, a distinct peak in the CO_2_ production rate at around 10 cm was not observed; the basic result was that CO_2_ production rates were largest between 0 and 10 cm. It is not clear whether this result is due to differences in soil characteristics or has its cause in the measurement method (the CO_2_ concentration at 50 cm was not measured at point B). However, what this result does indicate is that the patterns of CO_2_ production could be very different even though points A and B were only about 5 m apart. With the method that we used for this study, if the CO_2_ concentrations at various depths can be measured, CO_2_ production patterns can be measured at these depths relatively simply. Moreover, because it is currently possible to measure CO_2_ concentrations in soil relatively easily, we anticipate that it will be possible to evaluate the dynamics of soil CO_2_ production three-dimensionally by measuring CO_2_ concentrations at various points in the near future.

### Validity of the method

To test our analytical method, we performed a validation of the CO_2_ efflux from the soil. That is, we investigated how accurately CO_2_ effluxes from the soil could be estimated if the data assimilation were applied without using study data for CO_2_ effluxes. We found that although there were fluctuations in accuracy over time, the CO_2_ effluxes could be estimated relatively accurately. This seems to be an indirect confirmation that the diffusion equation for CO_2_ in the soil and its parameters have some level of validity. If the estimated diffusion coefficients and CO_2_ production rates used in the diffusion equation differed greatly from actual values, it is likely that these CO_2_ efflux estimates would have been different from the observed values.

The ideal validation would be to compare the estimated diffusion coefficients with the measured diffusion coefficients. To do that in practice, we would have to obtain the “actual” diffusion coefficients from laboratory experiments in which substantial soil samples are excavated from the field and moved intact to the laboratory. However, this process would invariably change the structure of the soils (e.g. the gas phase rate). In other words, it is very difficult to measure actual diffusion coefficients, even with destructive methods.

Using equations to calculate diffusion coefficients (e.g. Millington and Quirk), as done in previous studies, is the one of the best solutions to estimate diffusion coefficients in the field. We emphasize that this study does not negate that approach; rather, we propose a method to update the values of diffusion coefficients estimated by the Millington and Quirk equation through a Bayesian method that uses these coefficients as prior distributions. These values are then updated on the basis of observed CO_2_ concentration data and efflux data using a particle filtering method. This method also enables us to evaluate the uncertainties of the estimated values.

However, this method does not ensure the validity of the estimated diffusion coefficients and CO_2_ production rates. It only proposes the use of a Bayesian method to estimate the vertical profile of the CO_2_ production rate. Bayesian methods do not allow us to learn the “actual” values of the target parameters. However, the Bayesian method enables us to more closely approach the “actual” values by decreasing the variance of the posterior distribution of the values.

### CO_2_ efflux during snow-cover period

The estimated CO_2_ effluxes from the soil surface during the snow-cover period are comparable to those reported in previous studies. For example, Mariko et al. [33] and Mo et al. [19] estimated that the CO_2_ efflux during winter accounted for <15% and 10%, respectively, of the annual emission of Japanese temperate forests. Although the relative contribution of the snow-covered seasonal efflux to annual CO_2_ efflux has large variability even within the same ecosystem type [34], this would be indirect support for the validity of the estimated values of CO_2_ effluxes in this study.

There are a number of uncertainty factors for the values of CO_2_ efflux estimated in this study. The first is uncertainty due to the low reproducibility of soil CO_2_ concentrations near the soil surface. As can be seen from Fig. 1, estimates of CO_2_ concentrations at 0 cm during snow-cover periods were not particularly accurate, which may have had an effect on the estimated values of CO_2_ efflux from the soil surface during snow-cover periods. One possible cause of this is that the estimated diffusion coefficient at −1 cm (a diffusion coefficient that should be estimated to incorporate the diffusion coefficient of snow and the diffusion coefficient of air) was higher than the actual value. In fact, snow cover has a lower diffusion coefficient than air and acts as a kind of lid that may contribute to an increase in the CO_2_ concentration at 0 cm. This effect may not have been reproduced well in the model. Therefore, it may be that the estimated CO_2_ efflux during snow-cover periods was overestimated relative to actual efflux. However, the residence time of CO_2_ in snow seems to be several hours (no more than 1 day) as inferred from Yonemura et al. 2013 [18]. Therefore, the residence time of CO_2_ in snow should not have a large effect on the annual total efflux. A second uncertain factor is the influence of the sensors measuring the CO_2_ concentrations. These sensors may produce some heat and thus raise the temperature of the soil higher than it would otherwise be, particularly in winter periods; this also contributes to the possibility that the CO_2_ efflux was overestimated relative to the actual flux.

### Temperature sensitivity

An important aspect of this method is that we did not directly use soil temperature and water content data in the estimation of the CO_2_ production rate. Therefore, we were able to evaluate the relationship between soil temperature and CO_2_ production rate at each soil depth as a post hoc analysis.

The estimated Q_10_ values of the field data and experimental data were around the same range, averaging 2.28 and 2.33, respectively. This may be considered a crude and indirect validation of the estimated CO_2_ production rates by the particle filtering method, because the soils we tested were put through a 2 mm sieve, there was no cycling of organic matter and microorganisms in the soils, and the field data include root respiration in addition to microbial respiration [29]. However, we think that our efforts show that the estimated CO_2_ production rates are fairly realistic in spite of not using temperature and water content data directly.

There are two possible merits of this method. First, we can estimate the uncertainty of the estimated CO_2_ respiration rates and other relevant values. Second, we can improve the values estimated from empirical equations based on the diffusion process, such as the Millington and Quirk equation, by using CO_2_ concentration data and efflux data. Moreover, we can use the estimated CO_2_ production rates to improve models of soil organic carbon decomposition in future studies because the method does not rely directly on soil temperature and soil water content to estimate CO_2_ production rate. We believe that data assimilation methods hold great promise for studies of the relationship between soil respiration and environmental change.

## Supporting Information

S1 Fig Estimated CO_2_ production rates (mol m^–3^ day^–1^) at 50-day intervals between 100 and 450 days. The blue lines are the estimated average CO_2_ production rates. The gray areas are the 95% confidence intervals.
(TIF)

S2 Fig Estimated CO_2_ production rates (mol m^–3^ day^–1^) at 50-day intervals between 500 and 900 days. The blue lines are the estimated average CO_2_ production rates. The gray areas are the 95% confidence intervals.
(TIF)

S1 Sample Code Sample code of the particle filtering method. The sample code is written by R program. We confirmed the operation of the program by R version 2.15.3. This is only a sample code. Therefore, you have to change the codes according to your data, particularly the codes relevant to data-input.
(R) Click here for additional data file.
