# Supplementary figures and images for: Inversely Estimating the Vertical Profile of the Soil CO2 Production Rate in a Deciduous Broadleaf Forest Using a Particle Filtering Method
Source: PLoS One. 2015 Mar 20;10(3):e0119001. doi: 10.1371/journal.pone.0119001 (PMC4368638; doi:10.1371/journal.pone.0119001)

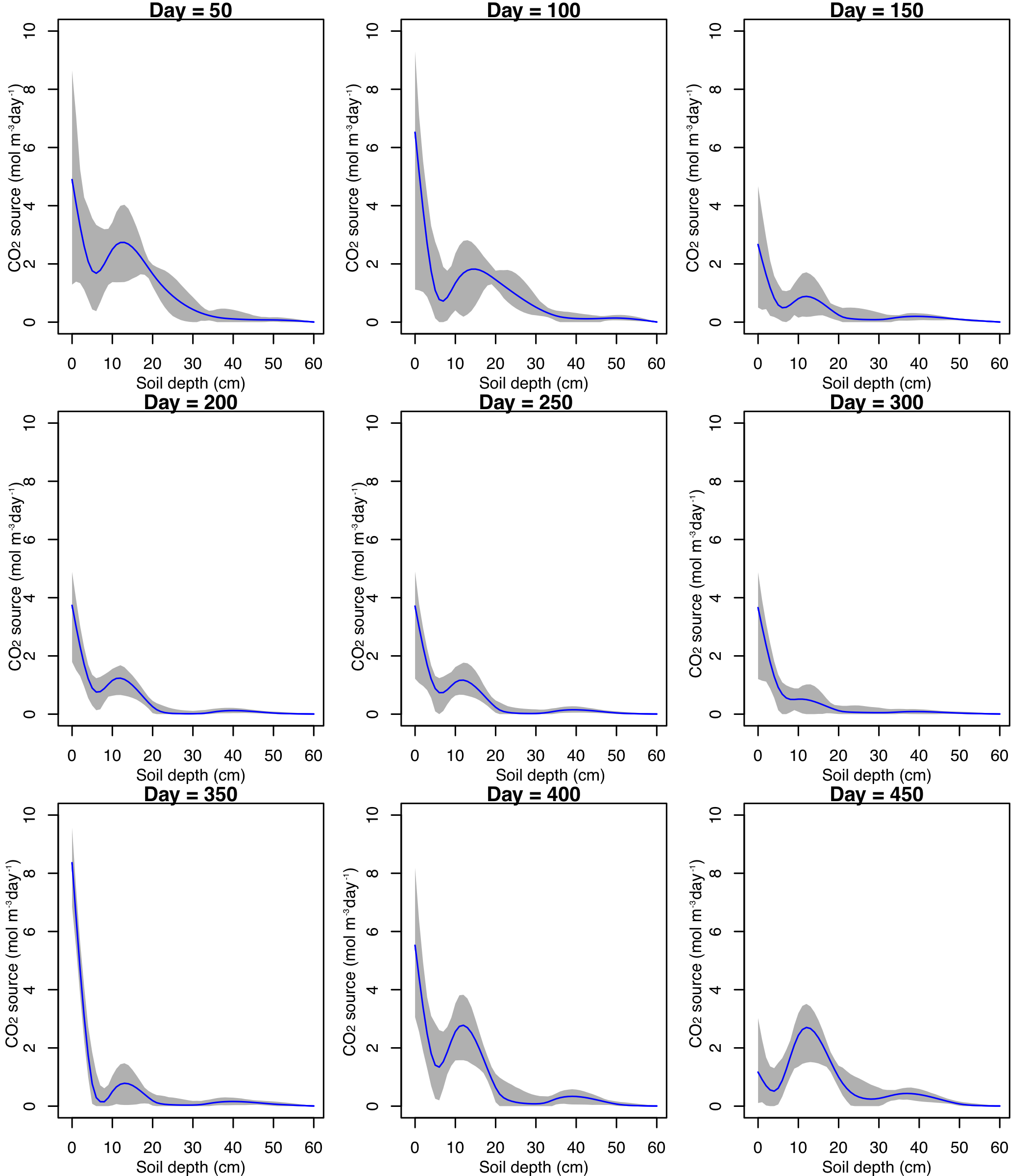

Supplement: S1 Fig — The blue lines are the estimated average CO2 production rates. The gray areas are the 95% confidence intervals. (TIF) [file pone.0119001.s001.tif]

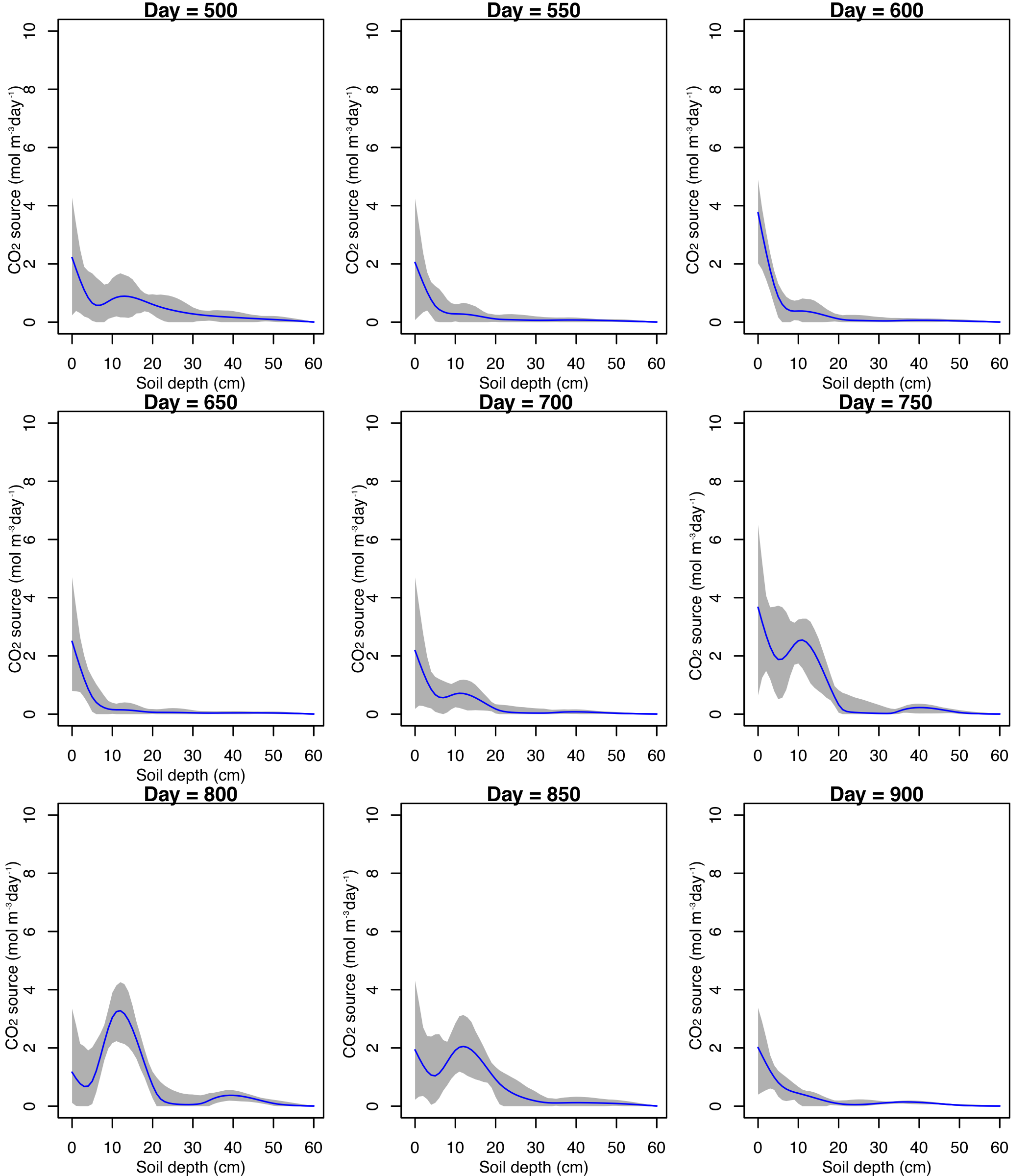

Supplement: S2 Fig — The blue lines are the estimated average CO2 production rates. The gray areas are the 95% confidence intervals. (TIF) [file pone.0119001.s002.tif]
